# Artemisinin relieves myocardial ischemia-reperfusion injury *via* modulating miR-29b-3p and hemicentin 1

**DOI:** 10.3389/fphar.2022.918966

**Published:** 2022-08-11

**Authors:** Junyu Han, Ziguan Zhang, Zhonghe Zhang, Shuyu Yang

**Affiliations:** ^1^ Department of Cardiology, The First Affiliated Hospital of Xiamen University, School of Medicine, Xiamen University, Xiamen, Fujian, China; ^2^ Xiamen Diabetes Institute, The First Affiliated Hospital of Xiamen University, School of Medicine, Xiamen University, Xiamen, Fujian, China

**Keywords:** myocardial ischemia-reperfusion, artemisinin, high throughput sequencing, miR-29b-3p, hemicentin 1, oxidative stress

## Abstract

**Objective:** To explore the impact of artemisinin (ARS) on myocardial ischemia-reperfusion (I/R) injury and the underlying mechanism.

**Methods:** Myocardial I/R rat model and cell model were used in this study. The cell viability, morphological changes, apoptosis, and oxidative stress were evaluated in cardiomyocytes H9c2 cells *in vitro* by using cell counting kit-8, microscope, flow cytometry, and commercial kits. High throughput sequencing is used to identify molecular targets of ARS on myocardial I/R injury, and then the gene-gene interaction network was constructed. MiR-29b-3p, hemicentin 1 (HMCN1), and apoptosis-related genes were tested by qRT-PCR and Western blotting. In the myocardial I/R rat model, echocardiography, (Triphenyl tetrazolium chloride) TTC staining, Hematoxylin-eosin (H&E) staining, Masson Trichrome staining, and TUNEL staining are applied to evaluate the protective effect of ARS on the myocardial injury.

**Results:** *In vitro*, we demonstrated that ARS alleviated H_2_O_2_-induced myocardial I/R injury, manifested by increased H9c2 viability, decreased pathological changes, apoptosis, and oxidative stress biomarker ROS, LDH, and CK-MB. Then, sequencing analysis revealed that miR-29b-3p/HMCN1 was the target of ARS for myocardial I/R injury. Notably, rescue experiments indicated that ARS inhibited myocardial I/R injury through targeted regulation miR-29b-3p/HMCN1. *In vivo*, we confirmed that ARS reduced myocardial injury, fibrosis, and apoptosis *via* modulation of miR-29b-3p/HMCN1.

**Conclusion:** This study demonstrated the functional role of the ARS/miR-29b-3p/HMCN1 axis in alleviating myocardial I/R injury, which provided a new direction for myocardial I/R injury therapy.

## Introduction

Globally, the morbidity and mortality of cardiovascular diseases remain high (Meng et al., 2018). Most of the patients who die of cardiovascular diseases are due to myocardial infarction (MI), resulting from insufficient blood supply to the heart (Neri and Riezzo 2017). Clinically, thrombolytic/fibrinolytic therapy and percutaneous coronary intervention can effectively restore the blood flow of the ischemic myocardium (Bagheri et al., 2016; Khan et al., 2018). However, reperfusion by the above methods might result in ischemia-reperfusion (I/R) injury (Sun et al., 2016). It is known that various pathophysiological factors are involved in myocardial I/R injuries, such as calcium overload, endothelial dysfunction, oxygen-free radical production, immune response, mitochondrial dysfunction, and apoptosis of cardiomyocytes (Matsui et al., 2007; Mokhtari-Zaer et al., 2018). Although significant advances have been made in understanding the mechanisms of I/R injury, the protective function of clinical therapies appears to be limited. Therefore, exploring the molecular events related to I/R injury and finding new targets to reduce I/R injury are urgently needed.

Artemisinin (ARS), a sesquiterpene endoperoxide, is mainly derived from Artemisia annua (Fang et al., 2019). ARS has been used as a first-line drug for the treatment of falciparum malaria because of its curative effect and low toxicity (Cao et al., 2019). With the in-depth research on ARS, it has been discovered that ARS has effective anti-malarial effects and has biological functions such as anti-tumor, anti-inflammatory, and immune regulation. For example, ARS induced apoptosis in colorectal cancer by restraining endoplasmic reticulum calcium ATPase activity (Frias et al., 2013). In an experimental model of hepatic fibrosis, ARS alleviated tissue damage and improved hepatic fibrosis by reducing the production of inflammatory cytokines (Lai et al., 2015). In MRL/lpr mice, ARS played a renal protective role by inhibiting B cell proliferation (Wu et al., 2016). In addition, there have been studies on the pharmacological characteristics of ARS in the anti-diabetes (Guo et al., 2018), anti-fungal (Gautam et al., 2011), and anti-viral effects (Efferth 2018). However, there is no report on the role of ARS on cardiac I/R injury.

In this study, we aimed to investigate the impacts of ARS on myocardial I/R injury, and its underlying mechanism. We found that ARS could be deemed as a potential agent for managing myocardial I/R *via* modulating the miR-29b-3p/HMCN1 axis.

## Materials and methods

### Animals and groups

A total of 50 male Sprague Dawley (SD) rats (250–300 g) were obtained from Shanghai Animal Laboratory Centre. SD rats were housed in a specific pathogen free animal room at room temperature of 25°C and relative humidity of 50%–60% in the First Affiliated Hospital of Xiamen University. All the animal experiments were conducted according to the Principles of Laboratory Animal Care and the Use of Laboratory Animals. The ethics committee of The First Affiliated Hospital of Xiamen University approved the study. SD rats were randomly divided into five groups with 10 rats in each group, Control group, I/R group (Model group), ARS group, ARS + miR-29b-3p inhibiror, and ARS + miR-29b-3p inhibitor + si-HMCN1 group.

### Construction of I/R model

The rats were first anesthetized with pentobarbital sodium (70 mg/kg). Then, the thoracotomy was performed to expose the heart. Next, the left coronary artery (LCA) was ligated and bound with 6/0 nylon monofilament for 30 min to induce ischemia. After occlusion for 30 min, the nylon monofilament was released, and reperfusion was performed for 2 h to obtain the I/R injury model. After 3 days, the treatment groups were intraperitoneally injected with artemisinin (10 mg/kg) or miR-29b-3p inhibitor (20 mg/kg) or si-HMCN1 (20 mg/kg), and model rats or control group were given normal saline for 10 consecutive days.

### Cell culture

The rat cardiomyocyte H9c2 cells (American Type Culture Collection) was cultured in DMEM in a favorable environment. To induce I/R injury and investigate the role of ARS on I/R, H9c2 cells were treated with 100 μM H_2_O_2_ or H_2_O_2_ plus ARS (Low (5 μM), Medium (20 μM), and High (40 μM) concentration) or miR-29b-3p inhibitor (20 nM) or si-HMCN1 (20 nM) for 12 h (Sun et al., 2016; Chen et al., 2018).

### Cell counting Kit-8

The H9c2 were inoculated onto 96-well plates and treated accordingly. The CCK-8 solution (Chengdu Biopurify Phytochemicals Ltd., China) was added and then incubated for 2 h. Finally, the activity of each group of cells was determined at a 450 nm optical density.

### Morphological observation

In short, H9c2 cells were treated with H_2_O_2_, ARS, and H_2_O_2_ + ARS, respectively. After culturing for 24 h, the cell morphology was observed under a inverted phase contrast microscope (Olympus, Japan).

### Flow cytometry

First, H9c2 was suspended in ice PBS and then incubated in buffer solution with FITC-labeled Annexin-V. Next, PI was added and stored on ice in the dark. Finally, the samples were immediately tested on the Flow cytometer (FACScan, BD Biosciences).

### Western blot

Protein isolation was carried out using cell lysis buffer and their concentrations were measured. Proteins were separated by 15% SDS-PAGE and then transferred onto PVDF membranes (Bio-Rad, United States). Blocked with 5% non-fat dry milk, the membranes were subjected to incubation with anti-Cleaved-caspase-3, anti-Cleaved-caspase-9, anti-HMCN1, and β-actin (1:1,000, Proteintech Group Inc., China), followed by secondary antibody. Finally, ECL reagents were adopted to visualize the protein.

### qRT-PCR

TRIZOL^©^ reagent (Invitrogen), TaqMan miRNA reverse transcription kit, and SYBR Green were adopted to extract sample RNA, synthesize cDNA and perform qRT-PCR, respectively. The primer sequences for this experiment were shown below: miR-29b-3p (NR_031837.1) sense, 5′-TAG​CAC​CAT​TTG​AAA​TCA​GTG​TT-3′ and antisense, 5′-CTC​TAC​AGC​TAT​ATT​GCC​AGC​CAC-3′; HMCN1 (XM_039090461.1) sense, 5′-GCC​ATC​AGT​GCC​ACC​GAA​TA-3′, and antisense 5′-AGA​CAT​GCT​AGG​GGT​AGG​GG-3′; β-actin (V01217.1) sense, 5′-GCA​AAT​GCT​TCT​AGG​CGG​AC-3′, and antisense, 5′-GCT​CCC​CAC​ACC​CAG​TAG​AA-3′.

### Biochemistry indicators detection

To detect ROS-induced cell injury, the treated H9c2 were co-cultured with Dichloro-dihydro-fluorescein diacetate (DCFH-DA in the dark for 40 min and then observed by scanning confocal microscopy. The creatine kinase-MB (CK-MB) and lactate dehydrogenase (LDH) were detected *via* the CK-MB isoenzyme assay kit and LDH Cytotoxicity Assay Kit (Roche, United States), respectively. Besides, the concentrations of interleukin (IL)-6 and TNF-ɑ were assessed using ELISA kits (Roche, Basel, Switzerland).

### Sequencing and data analysis

For the transcriptome libraries, the paired-end sequencing (125-bp reads) was executed. For QC, adapter sequences, the number of fuzzy bases (Ns), the 5′ and 3′ end low-quality sequences, and reads with quality scores below 20 were trimmed. After QC, clean reads were aligned to the reference genome (Rno6.0), compressed and sorted using SamTools, and counted with reference genome annotation (ENSEMBL) using FeatureCounts to obtain the original reads of the known genes. The DERNAs (false discovery rate (FDR) < 0.05 and biological coefficient variation (BCV) = 0.1 were then identified by the “edgeR.”

For the small RNA library, the single-end sequencing (50 bp) was executed. For raw sequencing reads, the quality was checked using FastQC. Briefly, adapter sequences and Ns were discarded. The average base quality (>15) was not less than 40%, and the length of the trimmed reads was between 17–35 nt. After QC, clean reads were aligned to the reference (Rno6.0), compressed and sorted using SamTools, and then counted with reference genome annotation (miRBase) using FeatureCounts to obtain the original reads of the known genes. “EdgeR” was used to identify the DE genes with BCV = 0.1 and FDR < 0.05.

### Network construction

The intersection of DERNAs (mRNA, lncRNA, and miRNA) of the two comparison groups were used to construct the network. LncBase v2, RNAInter, and miRWalk were adopted to predict the relationship between lncRNAs and miRNAs. MiRDB, DIANA microT_CDS, and TargetScan were adopted to predict the relationship between mRNAs and miRNAs. Then, the above predictions were integrated to establish a lncRNA-miRNA-mRNA network. Pearson correlation coefficients between DEmiRNAs and DEmRNAs were calculated to enhance the reliability.

### Echocardiography and myocardial function evaluation

First, the rats were anesthetized with 3% isoflurane and placed supine on a rat plate. Then, the acupuncture needles were inserted into the limbs and connected to the leads. Finally, the Vevo2100 imaging system (VisualSonics) was used to collect the two-dimensional cardiac ultrasound images of the rats. The catheter was inserted into the carotid artery for ultrasonic index detection and then advanced into the left ventricle. As a result, the interventricular septal thickness (IVS), left ventricular internal end-systolic diameter (LVIDs), and left ventricular posterior wall thickness (LVPW) were detected. In addition, the maximum rate of the rise of left ventricular pressure increase (+dp/dt max) and the maximum rate of the rise of left ventricular pressure decrease (−dp/dt max) were recorded for hemodynamic evaluation.

### 2,3,5-triphenyl tetrazolium chloride (TTC) staining

After reperfusion, the hearts were excised, and the tissue sections were stained with 1% TTC for 25 min. Then, they were placed in 10% formaldehyde for 2 days. Finally, when the infarct area appeared white, the infarct size was then calculated.

### Hematoxylin-eosin (H&E) staining

After the corresponding treatment, the myocardial tissues were fixed with formaldehyde (10%) for 24 h, tissues were placed in a 5% nitric acid decalcification solution for 3–5 days. After washing with water, routine dehydration, clearing, paraffin immersion, embedding, and sectioning, the tissue sections were stained with H&E. Finally, the pathological changes in myocardial tissues were observed under a microscope (FV1000, Olympus, Japan).

### Masson trichrome staining

First, slices of myocardial tissue were fixed in Bouin’s solution at 37°C. Then, after washing with distilled water, Mayer’s Hematoxylin and acidic Ponceau were stained for 10 min. Subsequently, the section was dissolved in a 1% phosphomolybdic acid aqueous solution, and after 10–15 min, the specimen was transferred to aniline blue and stained at 37°C for 20 min. Finally, the samples were rapidly dehydrated in 95% ethanol and then treated with hyaluronic acidification with dimethylbenzene.

### TUNEL staining

First, myocardial tissues were fixed with 4% formaldehyde and then washed in PBS containing proteinase K (20 μg/ml) at 37°C. Afterward, myocardial tissues were incubated overnight with the two (1 and 2, 1:10) TUNEL reagents. Finally, TUNEL staining was observed under an optical microscope (Olympus, Tokyo, Japan).

### Statistical analysis

SPSS21.0 was used to analyze the data in this study and data were presented as the mean ± SD. Each experiment was repeated ≥3 times. Differences between all multiple groups were compared using one- or two-way ANOVA and Tukey’s *post hoc* test. A *p* < 0.05 was regarded as statistically different.

## Results

### ARS suppresses H_2_O_2_-induced myocardial I/R injury

**FIGURE 1:**
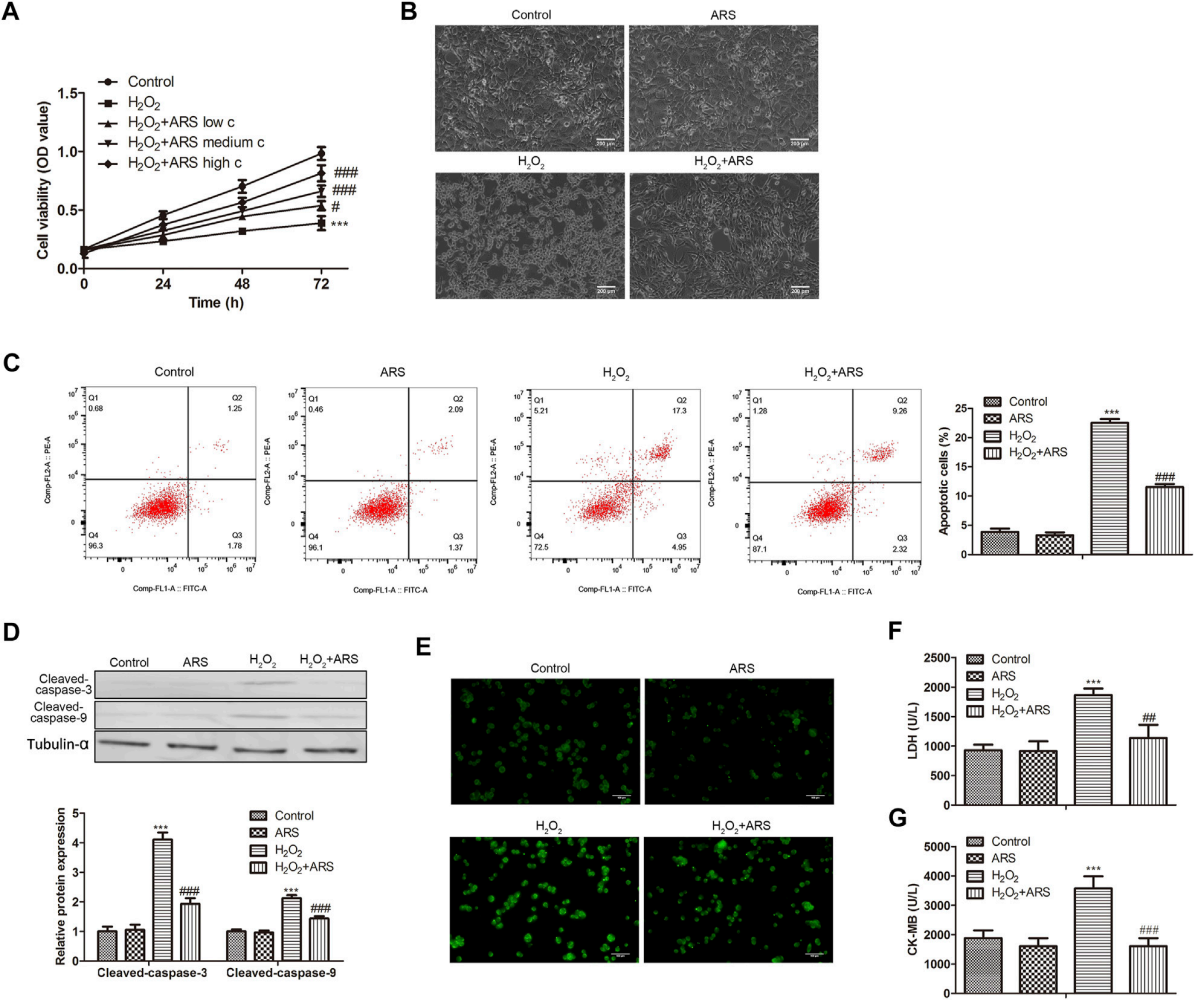
ARS suppresses H_2_O_2_-induced myocardial I/R injury. Proliferation, morphological changes, apoptosis, apoptosis-related protein, and oxidative stress biomarker in H9c2 cells *via* **(A)** CCK-8, **(B)** inverted phase contrast microscope (200 μm), **(C)** Flow cytometry, **(D)** Western blotting, **(E)** DCFH-DA (100 μm), **(F)** LDH Cytotoxicity Assay and **(G)** CK-MB isoenzyme assay, respectively. *N* = 3, ^***^ *p* < 0.001 vs. Control; ^#^ *p* < 0.5, ^###^ *p* < 0.001 vs. H_2_O_2_.

As presented in Figure 1A, H_2_O_2_ produced cell cytotoxicity and reduced H9c2 cell viability compared to the Control group. However, when ARS was added, it significantly reduced the toxicity of H_2_O_2_ to cells in a concentration-dependent pattern. Therefore, we selected the high concentration of ARS with the most significant cytotoxic remission for subsequent analysis. Under the microscope, we observed that H_2_O_2_ caused damage to the H9c2 cells, while ARS significantly alleviated the pathological injury induced by H_2_O_2_ (Figure 1B). In apoptotic analysis, the flow cytometry data revealed that the apoptotic cells in the ARS group were remarkably reduced compared to the H_2_O_2_ group (Figure 1C). Accordingly, pro-apoptotic proteins Cleaved-caspase-3 and Cleaved-caspase-9 were significantly decreased in H_2_O_2+_ARS group (Figure 1D). Furthermore, we detected ROS in the cells and found increased green fluorescence in the H_2_O_2_ group. On the contrary, when the H9c2 cells were co-incubated with ARS, the fluorescence intensity of H_2_O_2_ + ARS was sharply reduced (Figure 1E). Consistent with its effect on ROS, biochemistry indicators detection depicted that ARS significantly reduced the serum levels of markers LDH and CK-MB of myocardial injury (Figures 1F,G).

### ARS systematically altered the gene expression profile of H9c2 cells

**FIGURE 2:**
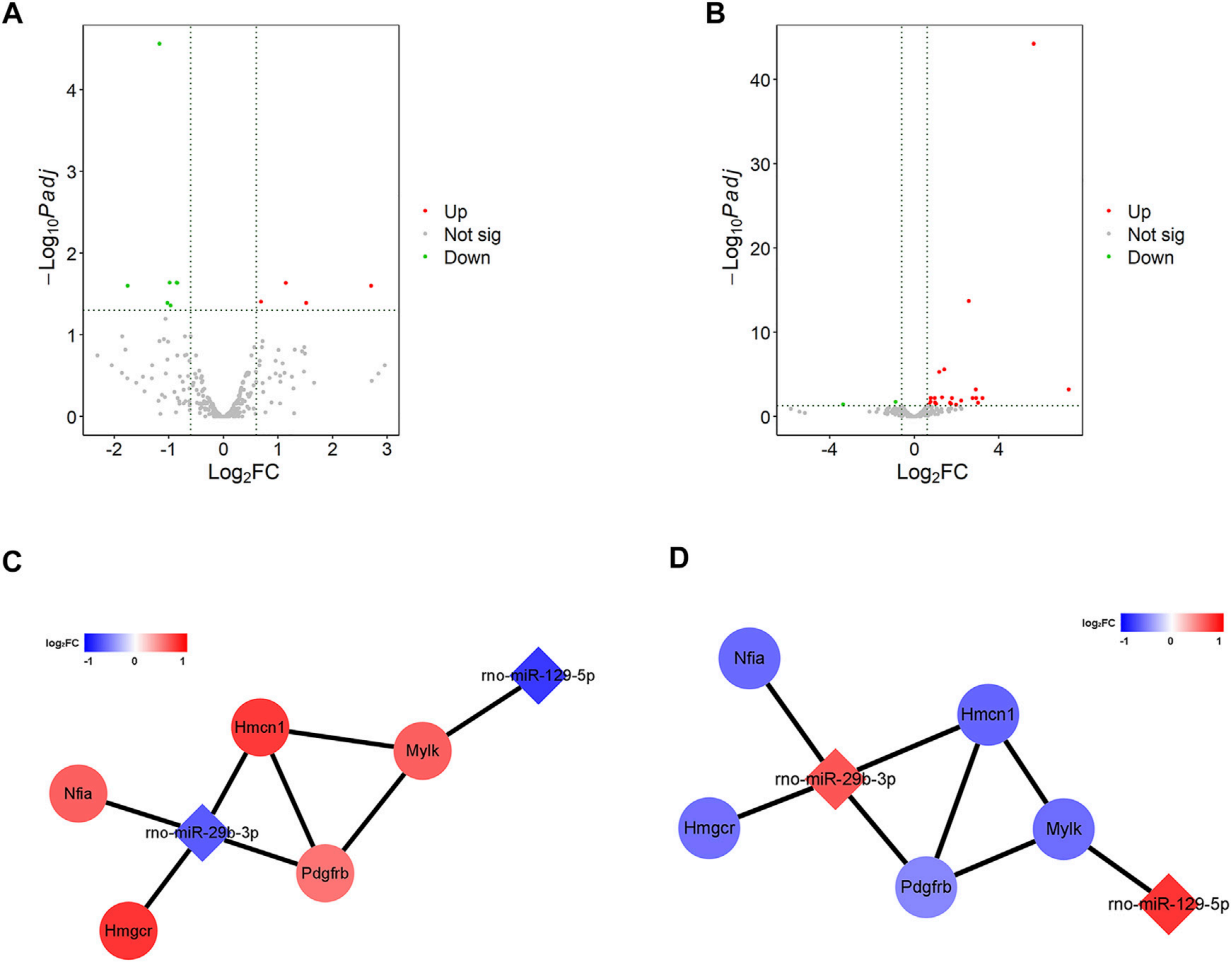
ARS systematically alters the gene expression profile of H9c2 cells. **(A**,**B)** The volcano plot of significant DEmiRNAs; **(C**,**D)** The miRNA-mRNA network.

To explore how ARS alleviates myocardial I/R injury, the changes of gene expression in H9c2 cells before and after ARS intervention were evaluated by sequencing. In the Control and the Model groups (Control vs. Model), there were 23 differential expressions of lncRNAs (DElncRNAs), with eight up-regulated and 15 down-regulated (Supplementary Figure S1A). There were 1,009 differential expressions of mRNAs (DEmRNAs), including 571 up-regulated and 438 down-regulated were found (Supplementary Figure S1B). There were 11 differential expressions of miRNAs (DEmiRNAs), of which four were up-regulated and seven were down-regulated (Figure 2A). In the Model and the Drug comparison groups (Model vs. ARS), there were 12 differential expressions of lncRNAs (DElncRNAs), with seven up-regulated and five down-regulated (Supplementary Figure S1C). There were 497 differential expressions of mRNAs (DEmRNAs), including 242 up-regulated and 255 down-regulated (Supplementary Figure S1D). There were 27 differential expressions of miRNAs (DEmiRNAs), of which 25 were up-regulated and two down-regulated (Figure 2B). Subsequently, the intersection of DE RNAs (mRNA, lncRNA, and miRNA) of the two comparison groups was taken to construct the competing endogenous RNA (ceRNA) network. Through LncBase v2, RNAInter, and miRWalk v2 database analysis, no miRNA-lncRNA pairs were found, while a total of 13 pairs of miRNA-mRNA were found through miRDB, DIANA, and TargetScan analysis. Furthermore, the interaction between genes was calculated online using STRING, and a total of five pairs of miRNA-mRNA were obtained that meet the expression trend (Figures 2C,D).

### MiR-29b-3p and HMCN1 are the target genes of ARS on myocardial I/R injury

**FIGURE 3:**
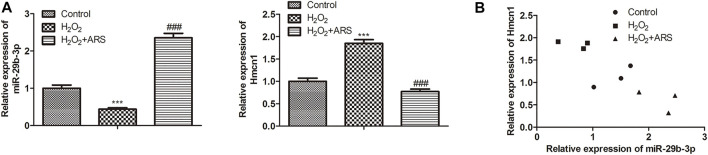
MiR-29b-3p and HMCN1 are the target genes of ARS. **(A)** MiR-29b-3p and HMCN1 was determined; **(B)** Pearson’s correlation analysis was applied to analyze the relationship between miR-29b-3p and HMCN1. *N* = 3, ^***^ *p* < 0.001 vs. Control; ^###^ *p* < 0.001 vs. H_2_O_2_.

**FIGURE 4:**
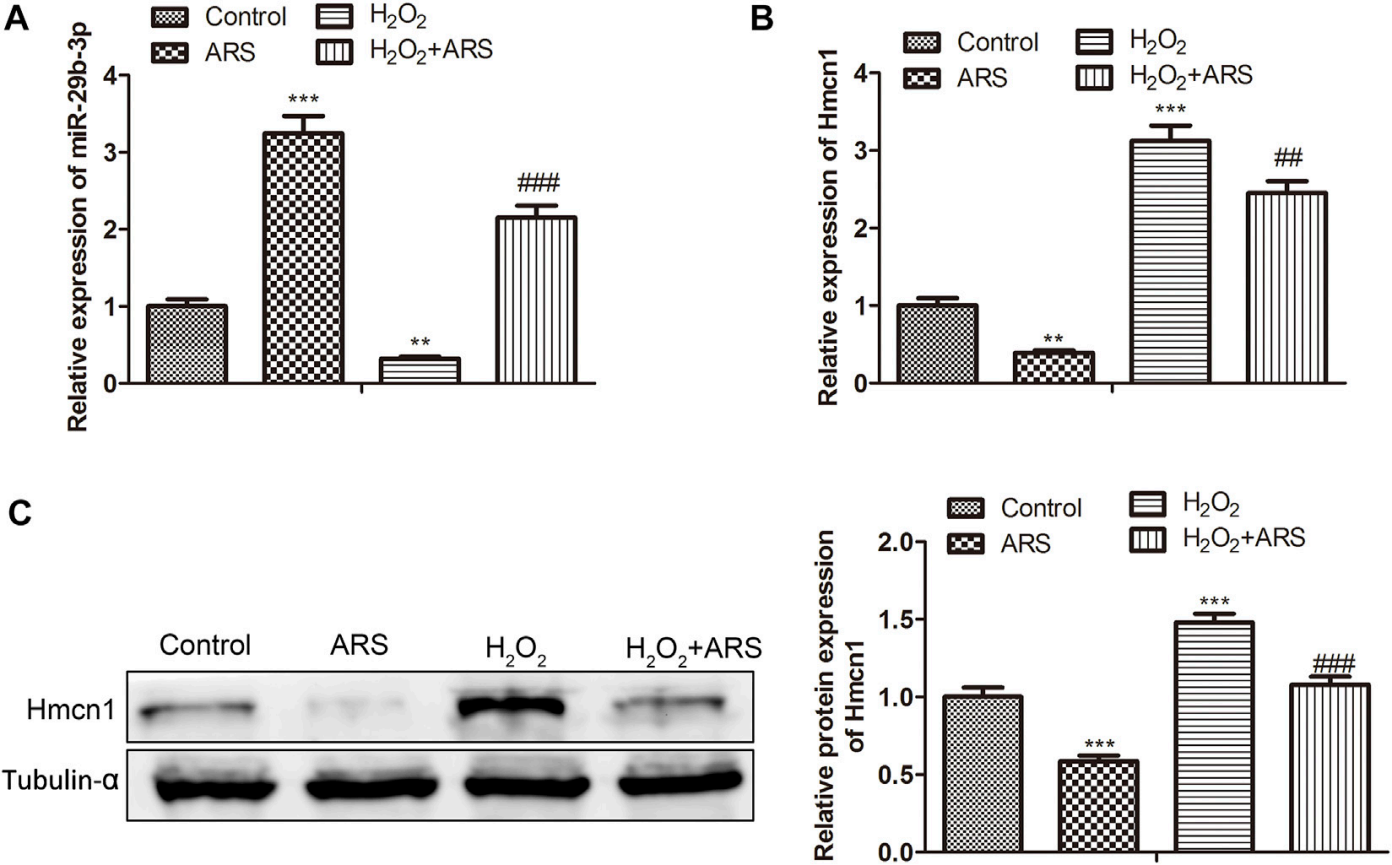
MiR-29b-3p and HMCN1 are the target genes of ARS on myocardial I/R injury. The expression of **(A)** miR-29b-3p and **(B)** HMCN1 in indicated groups tested using qRT-PCR; **(C)** Protein expression of HMCN1 evaluated *via* Western blotting. *N* = 3, ^**^ *p* < 0.01, ^***^ *p* < 0.001 vs. Control; ^##^ *p* < 0.01, ^###^ *p* < 0.001 vs. ARS or H_2_O_2_.

In the miRNA-mRNA network, miR-29b-3p and HMCN1 not only had higher node degrees but also had higher miRNA-mRNA pairs. Therefore, miR-29b-3p and HMCN1 were selected as the hub genes for further analysis, and their expression patterns were shown in Figure 3A. Briefly, ARS induced miR-29b-3p expression and conversely inhibited the HMCN1 level. Consistently, Pearson’s correlation analysis depicted that miR-29b-3p and HMCN1 were negatively correlated (Figure 3B). To further verify that miR-29b-3p and HMCN1 are the target genes of ARS, the changes of these two genes in H9c2 cells were examined. As expected, qRT-PCR data showed that H_2_O_2_ inhibited the miR-29b-3p expression and promoted HMCN1 expression. After ARS intervention, the expression of the above two genes was significantly reversed (Figures 4A,B). Consistently, we observed that ARS evidently inhibited the expression of HMCN1 protein (Figure 4C).

### ARS suppresses H_2_O_2_-induced myocardial I/R injury through regulation of miR-29b-3p/HMCN1 *in vitro*

**FIGURE 5:**
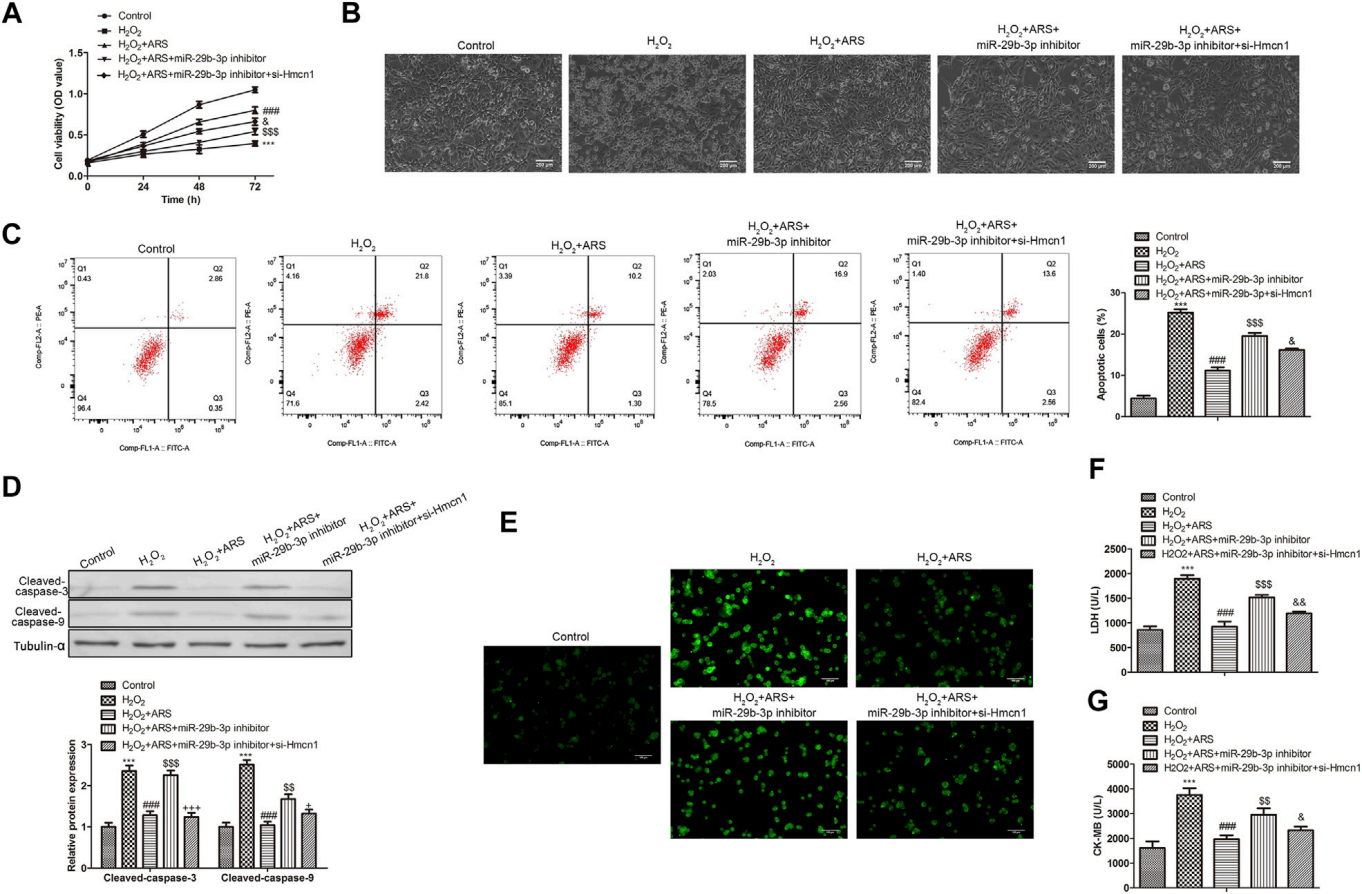
ARS suppresses myocardial I/R injury through regulating MiR-29b-3p/HMCN1. Proliferation, morphological changes, apoptosis, apoptosis-related protein and oxidative stress biomarker of H9c2 cells observed *via* **(A)** CCK-8, **(B)** inverted phase contrast microscope (200 μm), **(C)** Flow cytometry, **(D)** Western blotting, **(E)** DCFH-DA (100 μm), **(F)** LDH Cytotoxicity Assay and **(G)** CK-MB isoenzyme assay, respectively. *N* = 3, ^***^ *p* < 0.001 vs. Control; ^###^ *p* < 0.001 vs. H_2_O_2_; ^$$^ *p* < 0.01, ^$$$^ *p* < 0.001 vs. H_2_O_2_ *+* ARS; ^&^ *p* < 0.05, ^&&&^ *p* < 0.001 vs. H_2_O_2_ *+* ARS + miR-29b-3p inhibitor.

To further evaluate whether ARS executed its function *via* the miR-29b-3p/HMCN1 axis, rescue assays were performed. As shown in the CCK-8 results in Figure 5A, the miR-29b-3p inhibitor decreased the inhibitory effect of ARS. However, when H9c2 cells were co-transfected with si-HMCN1, the cell activity in H_2_O_2_ + ARS + miR-29b-3p inhibitor + si-HMCN1 group was significantly elevated compared to H_2_O_2_ + ARS + miR-29b-3p inhibitor group. Similarly, under the microscope, it appeared that the miR-29b-3p inhibitor enhanced the pathological damage of H9c2, while si-HMCN1 alleviated the cell damage caused by the miR-29b-3p inhibitor (Figure 5B). Flow cytometry and Western blotting analysis showed that ARS influenced cell apoptosis by regulating miR-29b-3p/HMCN1 (Figures 5C,D). Furthermore, we observed that the ROS level in the H_2_O_2_ + ARS + miR-29b-3p inhibitor group was significantly higher than that in the H_2_O_2_ + ARS + miR-29b-3p inhibitor + si-HMCN1 group (Figure 5E). Meanwhile, compared with the H_2_O_2_ + ARS + miR-29b-3p inhibitor group, the content of myocardial injury biomarkers (LDH and CK-MB) in H_2_O_2_ + ARS + miR-29b-3p inhibitor + si-HMCN1 group was also remarkably decreased (Figures 5F,G).

### ARS suppresses myocardial I/R injury by regulating miR-29b-3p/HMCN1 *in vivo*

**FIGURE 6:**
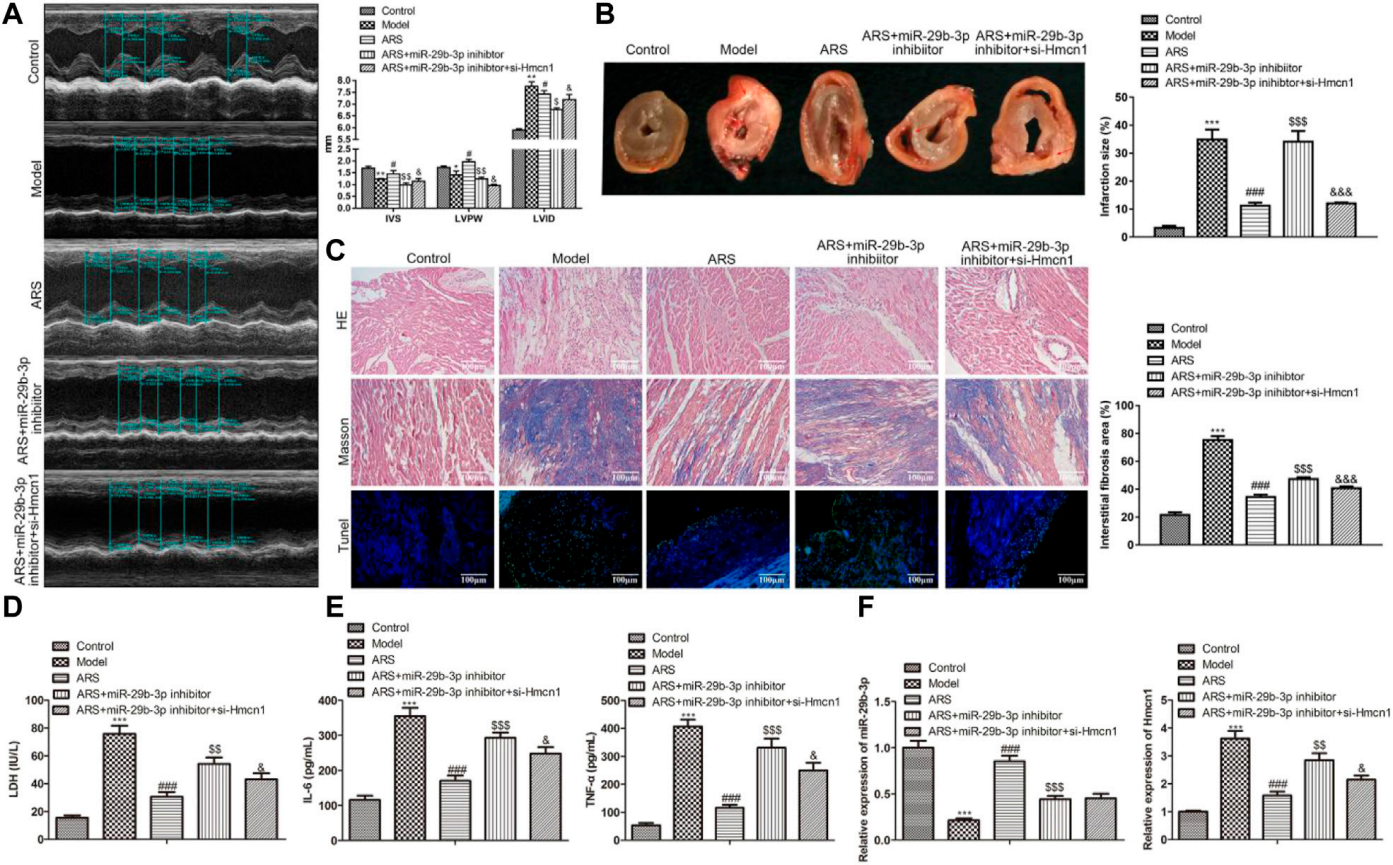
ARS suppresses myocardial I/R injury through regulating miR-29b-3p/HMCN1 in vivo.

We have demonstrated *in vitro* that ARS inhibited myocardial injury through the regulation of miR-29b-3p/HMCN1. To confirm this finding, we conducted *in vivo* studies. The cardiac function-related parameters revealed that compared with the Model group, IVS and LVIDs increased, whereas LVPW decreased significantly in the ARS treatment group. When miR-29b-3p inhibitor was added, IVS and LVIDs were relatively reduced, while LVPW was relatively increased. Interestingly, the co-transfection of si-HMCN1 reversed the regulation of miR-29b-3p inhibitor on these parameters (Figure 6A). Next, we observed the infarct size of the heart in each group after treatment under different conditions. The results showed that the myocardial infarction size in ARS + miR-29b-3p inhibitor + si-HMCN1 group was significantly smaller than that in the ARS + miR-29b-3p inhibitor group (Figure 6B). In addition, we observed the morphological changes in myocardial tissues in each group. As expected, the number of lesions, fibrosis, and apoptotic cells in the ARS + miR-29b-3p inhibitor was significantly increased compared with the ARS group. On the contrary, we observed significant improvement in these pathological changes in the ARS + miR-29b-3p inhibitor + si-HMCN1 group after adding si-HMCN1 (Figure 6C). Meanwhile, we also observed that LDH, IL-6, and TNF-ɑ in the ARS + miR-29b-3p inhibitor + si-HMCN1 group were relatively reduced compared with the ARS + miR-29b-3p inhibitor group (Figures 6D,E). Consistently, the expression trends of miR-29b-3p and si-HMCN1 in each group showed corresponding changes (Figure 6F).

## Discussion

Recovery of blood supply and re-oxygenation can lead to tissue injury, known as I/R injury (Schulman et al., 2002). Wang et al. (2020) found that artemisinin-treated myocardial I/R rats showed mild myocardial I/R injury (Wang et al., 2020). Additionally, art can attenuate MPP^+^ - induced cytotoxicity in human neuroblastoma cells (SH-SY5Y) (Yan et al., 2021). Similarly, our analysis based on a rat I/R injury model in this study showed that ARS alleviated I/R-induced cytotoxicity and alleviated pathological damage and fibrosis of the myocardial structure. It was known that one of the main manifestations of myocardial I/R injury is cell apoptosis (Yao et al., 2019), and the expression of vital apoptotic factors such as caspase-3 and caspase-9 increased significantly during I/R (Liu and Li 2020). Consistent with previous studies, we found that ARS reduced apoptosis in H9c2 cells and myocardial tissues. Correspondingly, ARS also reduced the protein expression of caspase-3 and caspase-9 in H9c2 cells, suggesting that reducing apoptosis is a mechanism of ARS for alleviating I/R injury. Additionally, oxidative stress is another primary mechanism involved in myocardial I/R (Rodrigo et al., 2013). In the report by Broskova and Tanwar et al., curcumin relieved myocardial I/R injury by eliminating ROS and reducing the leakage of LDH (Tanwar et al., 2010; Brosková et al., 2013). ART could significantly improve the viability, and reduce oxidative stress damage and apoptosis of MPP^+^-treated SH-SY5Y cells (Yan et al., 2021). Consistent with its effect on oxidative stress, ARS reduced the content of ROS and LDH in H29c cells, suggesting that ARS could reduce I/R injury.

MicroRNAs (miRNAs) can inhibit mRNA translation or activate mRNA degradation, thereby mediating gene silencing (Pei et al., 2020). MiRNAs exert an important effect on cell proliferation, survival, and other regulatory processes, and their abnormal expression is associated with the occurrence and development of various diseases, including heart disease (Fiedler and Thum 2013; Melman et al., 2015). In recent years, some miRNAs have been found to involve the process of I/R injury, including miR-328 (Lu et al., 2010), miR-182 (Zhao et al., 2019), and miR-378 (Ding et al., 2020). The miR-29b-3p has recently been found to play roles in cancer, inflammation, and cardiac fibrosis. For example, miR-29b-3p delayed cancer progression in bladder cancer by inhibiting pathological proliferation and invasion (Ding et al., 2018). In osteoarthritis, miR-29b-3p can directly bind to progranulin to accelerate the progression of inflammation (Chen et al., 2017). In a mouse model of chronic kidney disease, high expression of miR-29b-3p has been shown to reduce myocardial fibrosis (Drummond et al., 2018). MiR-29b-3p can aggravate I/R injury in myocardial (H9c2) cells by repressing PTX3 expression (He and Yan 2021). MiR-29b-3p can target the down-regulation of cIAP-1 expression in senescent cardiomyocytes and aggravate hypoxia/reoxygenation (H/R) - induced myocardial injury (Zhang et al., 2019). Here, in this study, we found that miR-29b-3p was an effective target for ARS in the treatment of I/R through high-throughput sequencing. Furthermore, molecular biological data from both *in vitro* and *in vivo* studies confirmed that low expression of miR-29b-3p contributed to the deterioration of myocardial I/R injury, increased pathological change, fibrosis, apoptosis, and oxidative stress.

Hemicentin 1 (HMCN1), also known as fibulin-6, was first discovered in *Caenorhabditis elegans* (Liu et al., 2019). HMCN1 is related to hemidesmosome structure and participates in extracellular adhesion, forming cell-to-cell and cell basement membrane adhesion, keeping cells together, and maintaining the integrity of tissues and organs (Xu and Vogel 2011). Studies have shown that abnormal expression of HMCN1 is associated with age-related macular degeneration (Pras et al., 2015), tumorigenesis (Liu et al., 2019), Fraser syndrome (Carney et al., 2010), and cell fibrosis (Chowdhury et al., 2017) and glomerular disease (Toffoli et al., 2018). Little was known about the characteristics of HMCN1 in myocardial I/R injury. Here, our data confirmed that HMCN1 was involved in the biological process of reducing I/R injury upon ARS treatment. HMCN1is a target of miR-29b-3p and that HMCN1 knockout helps ameliorate I/R injury.

## Conclusion

In summary, we reported that ARS intervention reduced myocardial I/R injury. Furthermore, the observed beneficial effects of ARS are related to the miR-29b-3p/HMCN1 axis. The finding of this new mechanism will help develop new and better treatment options for patients with myocardial I/R injury.

## Data Availability

The datasets presented in this study can be found in online repositories. The names of the repository/repositories and accession number(s) can be found below: https://www.ncbi.nlm.nih.gov/bioproject/PRJNA846860.
