# Corrigendum: Artemisinin relieves myocardial ischemia-reperfusion injury *via* modulating miR-29b-3p and hemicentin 1

**DOI:** 10.3389/fphar.2023.1169734

**Published:** 2023-03-01

**Authors:** Junyu Han, Ziguan Zhang, Zhonghe Zhang, Shuyu Yang

**Affiliations:** ^1^ Department of Cardiology, The First Affiliated Hospital of Xiamen University, School of Medicine, Xiamen University, Xiamen, Fujian, China; ^2^ Xiamen Diabetes Institute, The First Affiliated Hospital of Xiamen University, School of Medicine, Xiamen University, Xiamen, Fujian, China

**Keywords:** myocardial ischemia-reperfusion, artemisinin, high throughput sequencing, miR-29b-3p, hemicentin 1, oxidative stress

In the published article, there was an error in **Figure 6** as published. The corrected Figure 6 and its caption appear below.

**FIGURE 6:**
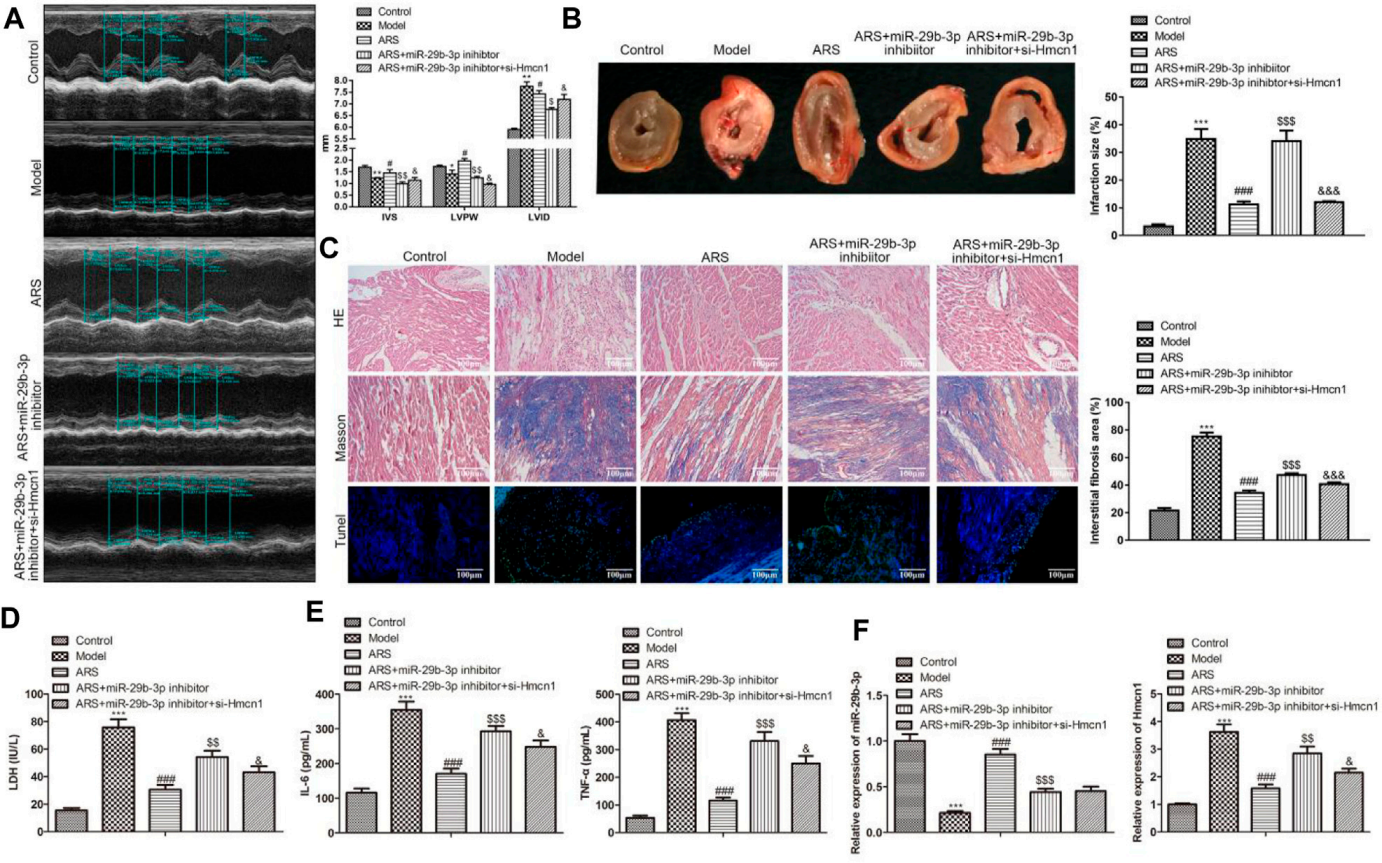
ARS suppresses myocardial I/R injury through regulating miR-29b-3p/HMCN1 in vivo.

The authors apologize for this error and state that this does not change the scientific conclusions of the article in any way. The original article has been updated.

