# Supplementary figures and images for: Artemisinin relieves myocardial ischemia-reperfusion injury via modulating miR-29b-3p and hemicentin 1
Source: Front Pharmacol. 2022 Aug 11;13:918966. doi: 10.3389/fphar.2022.918966 (PMC9403756; doi:10.3389/fphar.2022.918966)

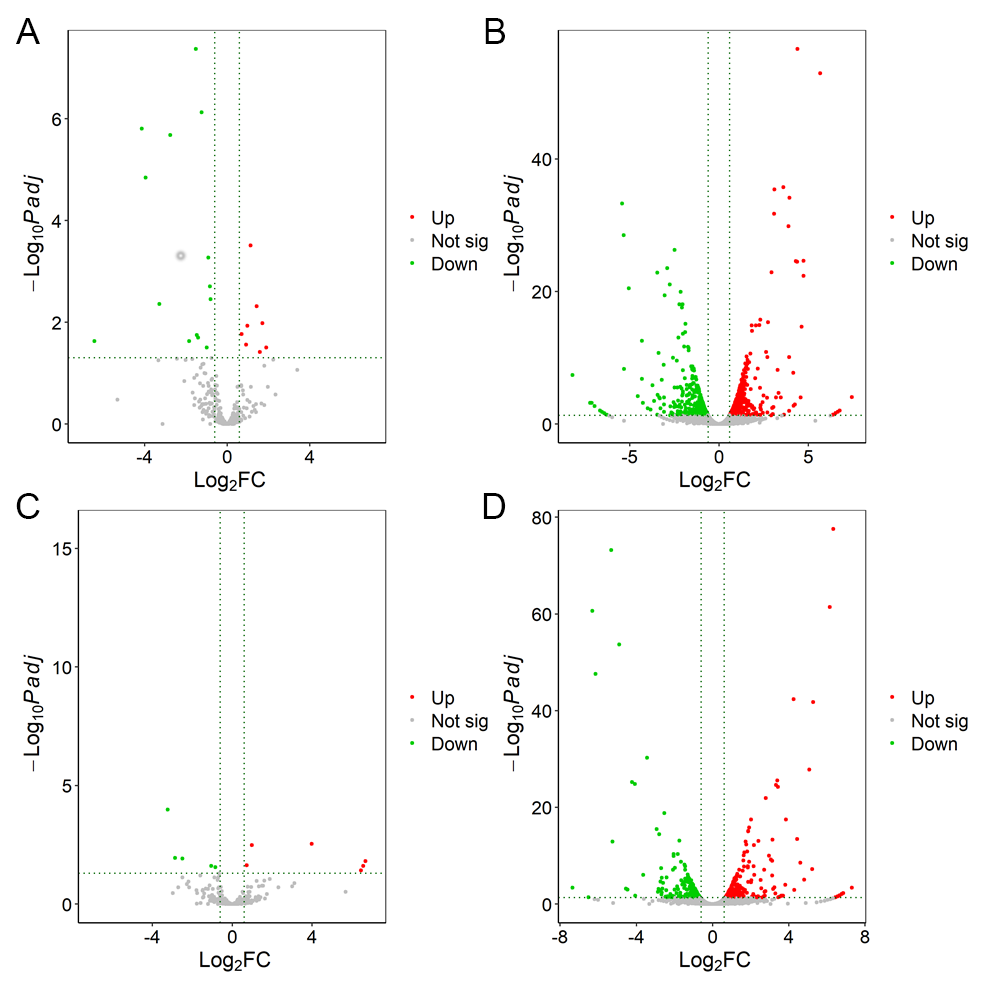

Supplement: Supplementary file 1 [file Image1.TIF]
