# Supplementary material for: Artemisinin relieves myocardial ischemia-reperfusion injury via modulating miR-29b-3p and hemicentin 1
Source: Front Pharmacol. 2022 Aug 11;13:918966. doi: 10.3389/fphar.2022.918966 (PMC9403756; doi:10.3389/fphar.2022.918966)

ceRNA network

Figure 2C

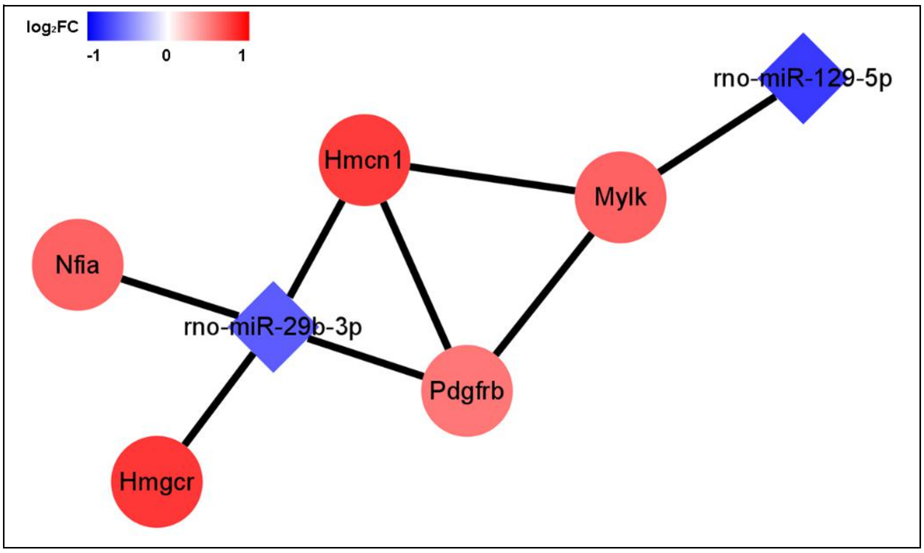

Figure 2D

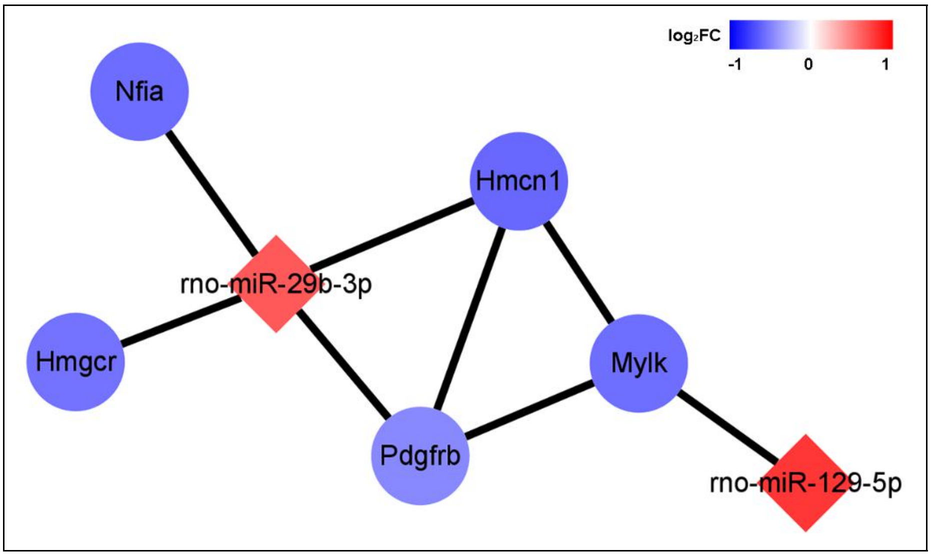

detect ROS

Figure 1E

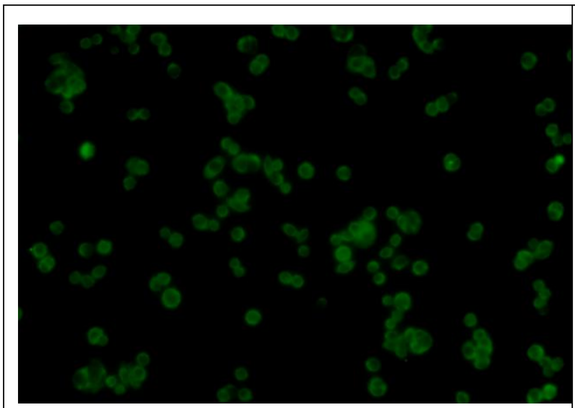	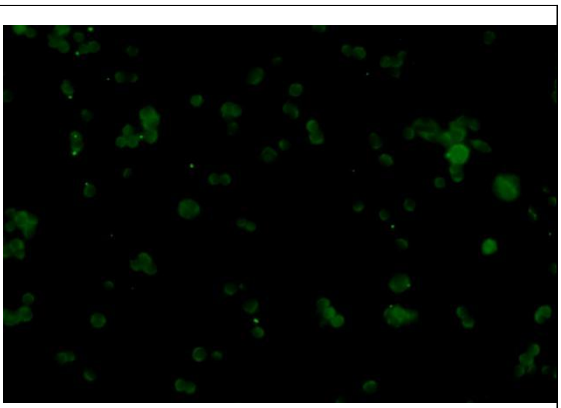
**Control**	**ARS**
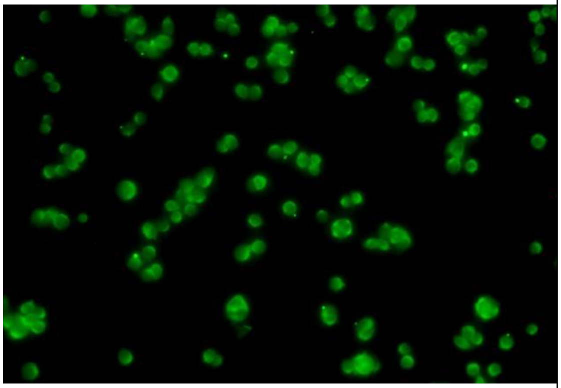	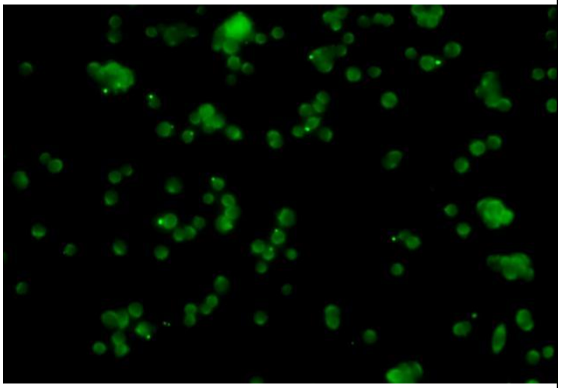
**H2O2**	**H2O2+ARS**

detect ROS

Figure 5E

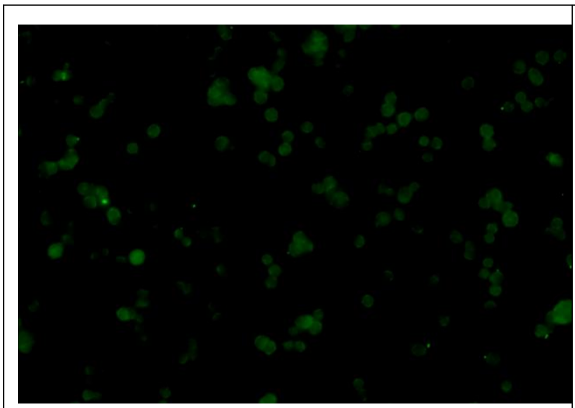	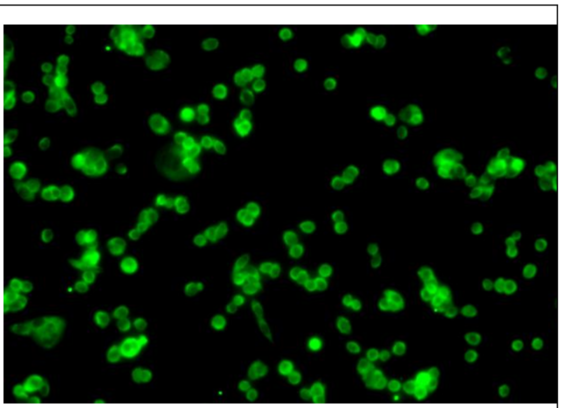
**Control**	**H2O2**
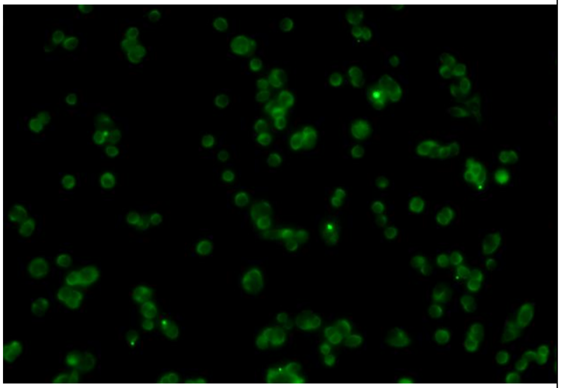	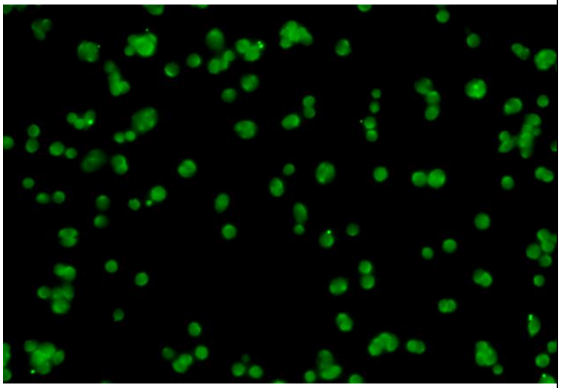
**H2O2+ARS**	**H2O2+ARS+miR-29b-3p inhibitor**
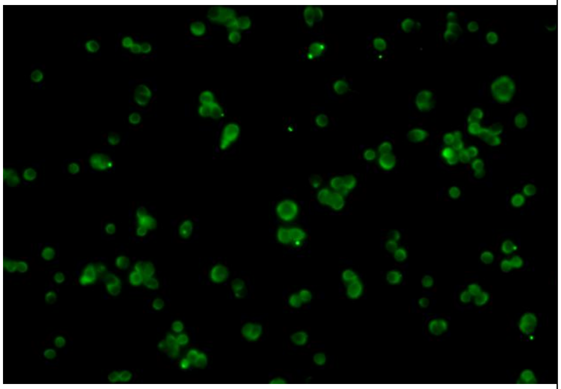	
**H2O2+ARS+miR-29b-3p inhibitor+si-Hmcn1**	

Echocardiography

Figure 6A

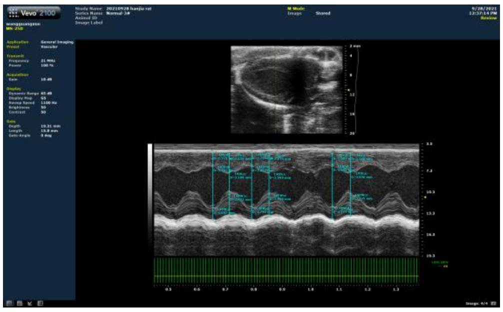	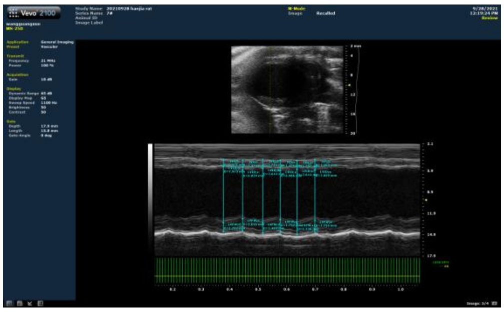
**Control**	**Model**
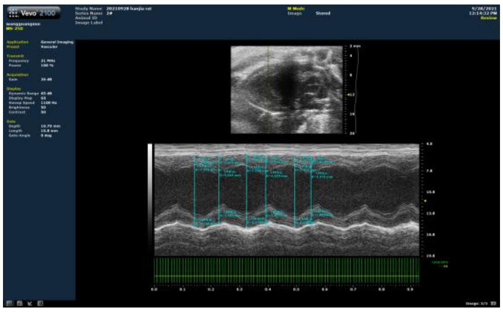	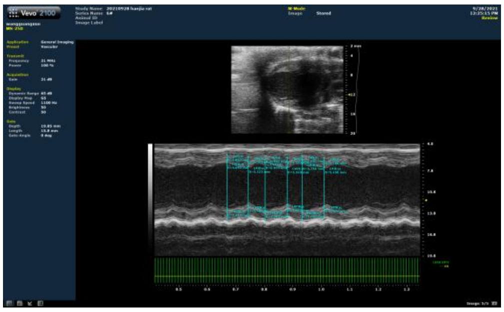
**ARS**	**ars+miR-29b-3p-inhibitor**
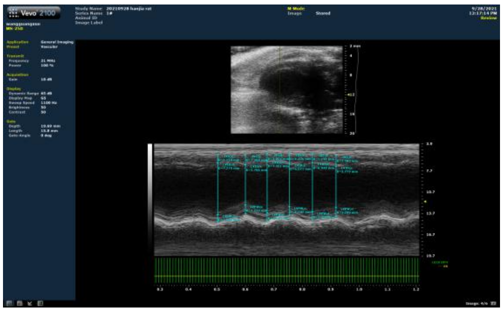	
**ars+miR-29b-3p-inhibitor+si-hmcn<br>1**	

Flow cytometry

Figure 1C

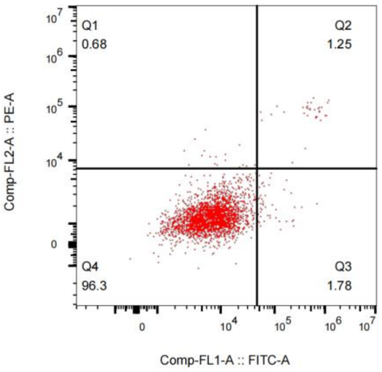

Control

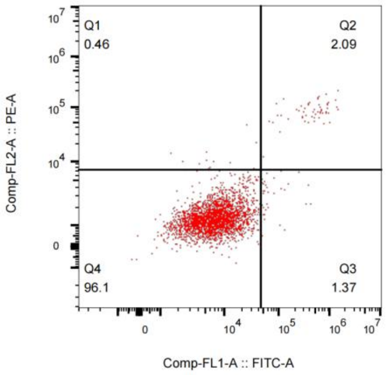

ARS

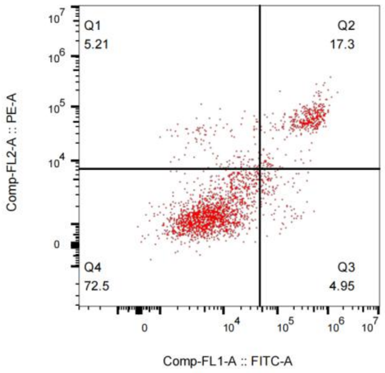

H2O2

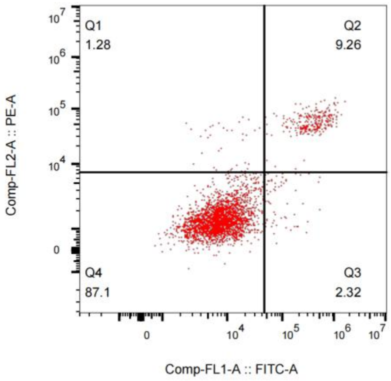

H2O2+ARS

Flow cytometry

Figure 5C

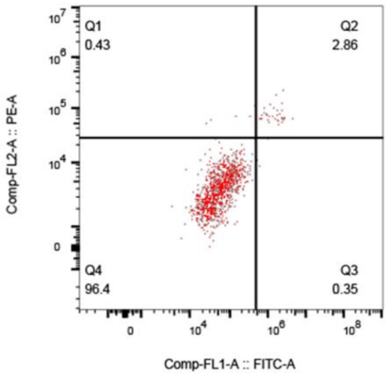

Control

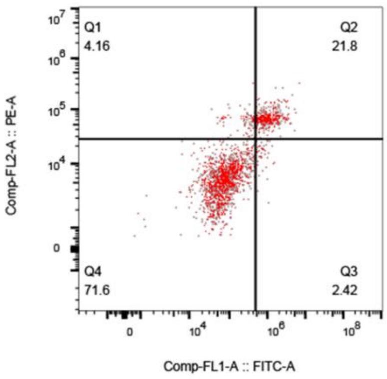

H_2O_2

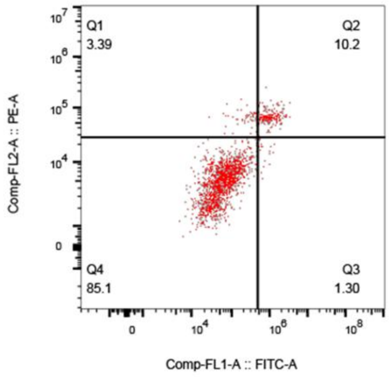

H2O2+ARS

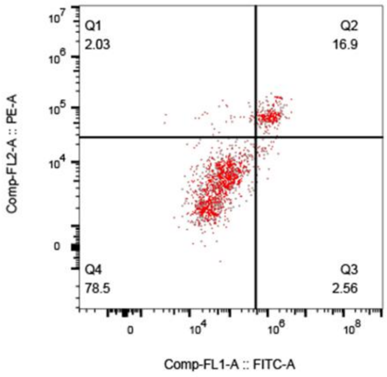

H2O2 +ARS+ miR-29b-3p inhibitor

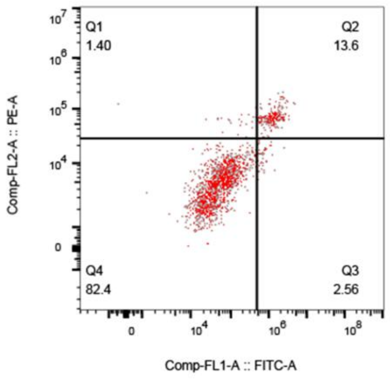

H2O2+ARS+ miR-29b-3p inhibitor+si-Hmcn1

gene expression profile

Figure 2A

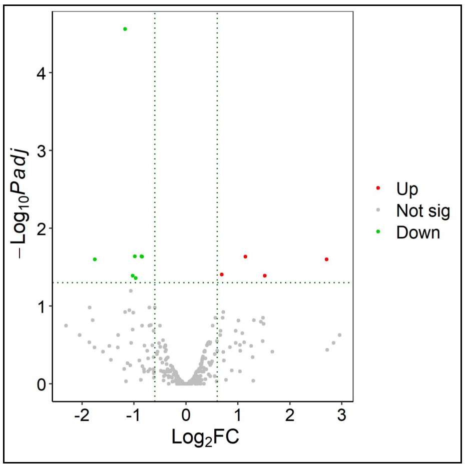

gene expression profile

Figure 2B

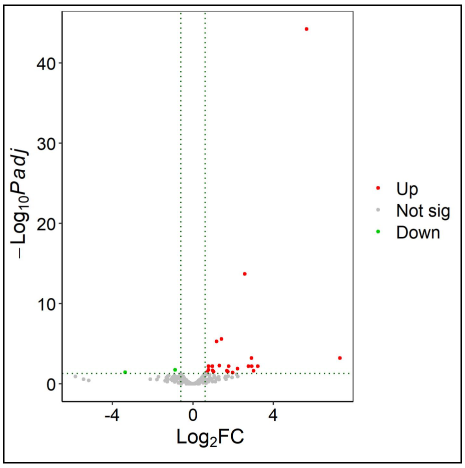

HE TUNEL Masson

Figure 6C

HE	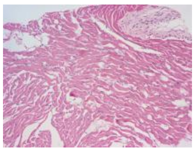	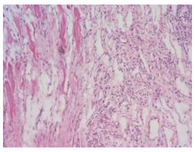	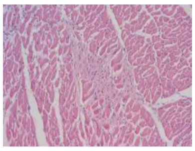	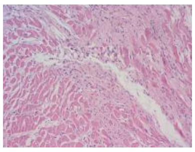	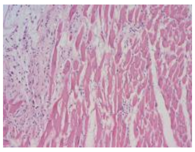
Ma sso n	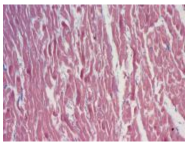	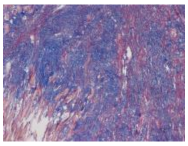	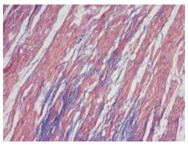	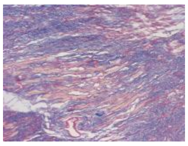	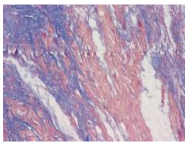
TU NE L	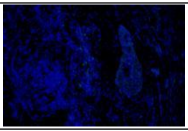	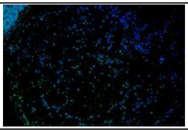	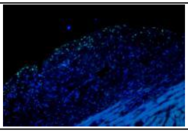	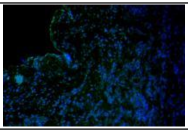	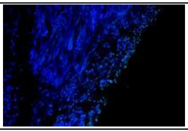
	Control	model	ARS	ARS+miR-29 b-3p inhibitor	ARS+miR-29 b-3p inhibitor+si-Hmcn1

Morphological observation

Figure 1B

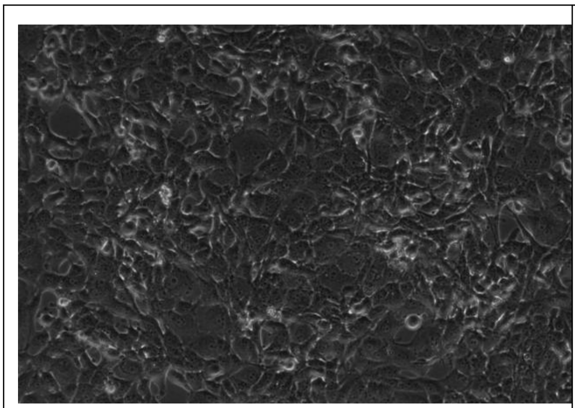	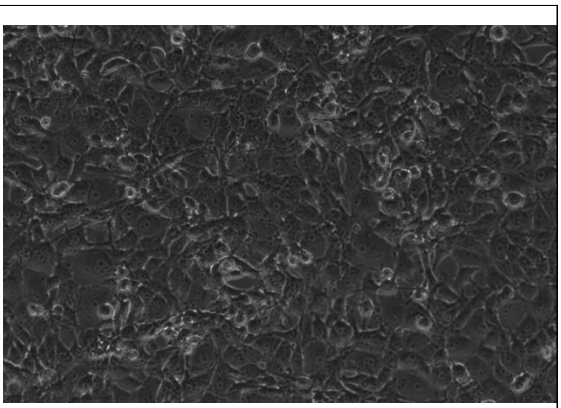
**Control**	**ARS**
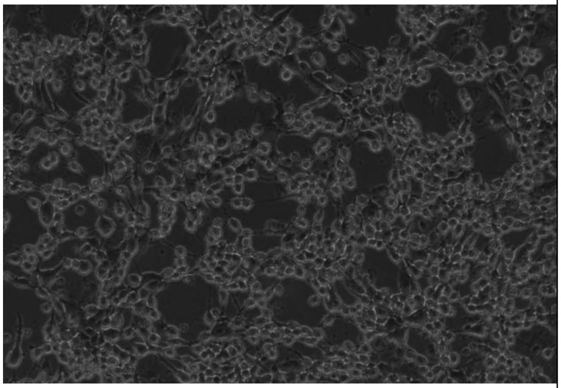	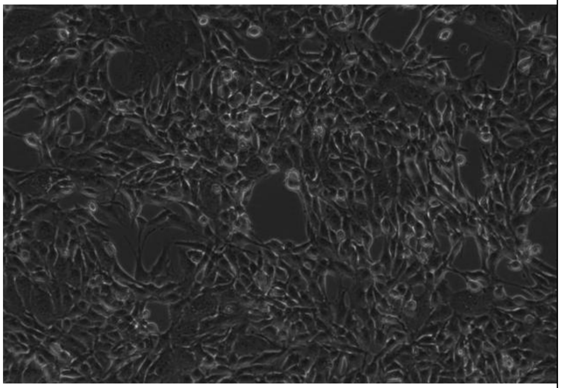
**H_2O_2**	**H2O2+ARS**

Morphological observation

Figure 5B

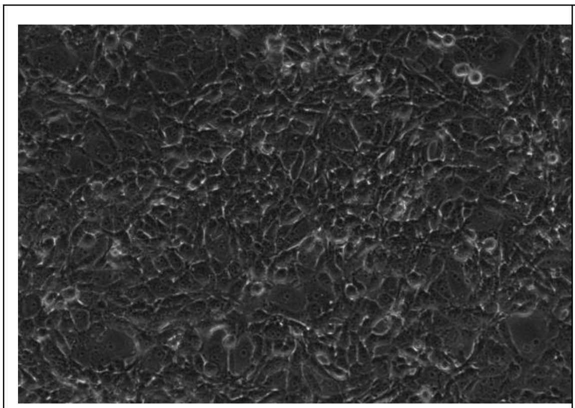	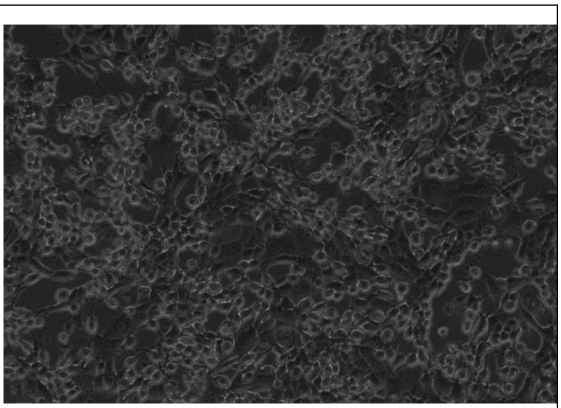
**Control**	**H_2O_2**
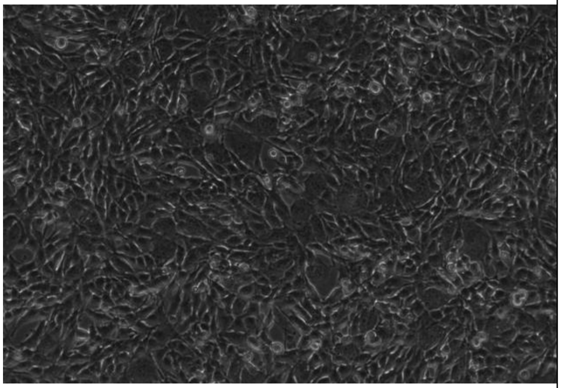	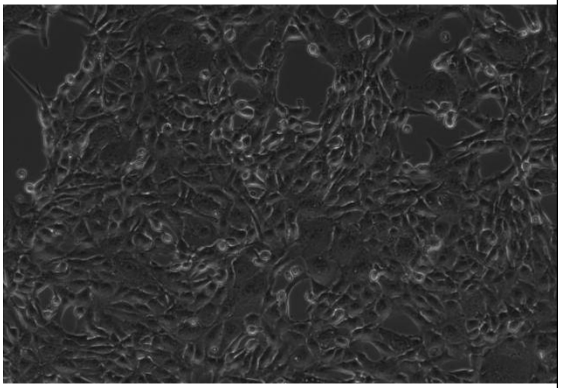
**H2O2+ARS**	**H2O2+ARS+miR-29b-3p inhibitor**
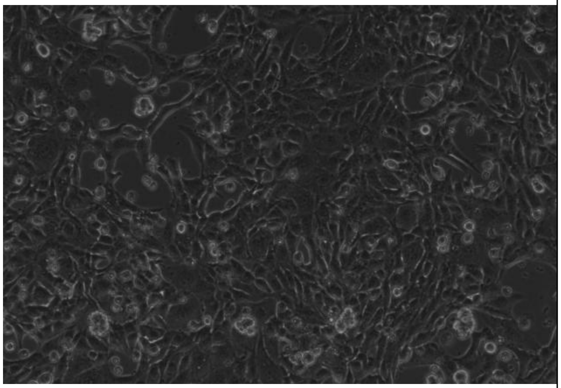	
**H2O2+ARS+miR-29b-3p inhibitor+Hmcn1**	

TTC

Figure 6B

**Control**	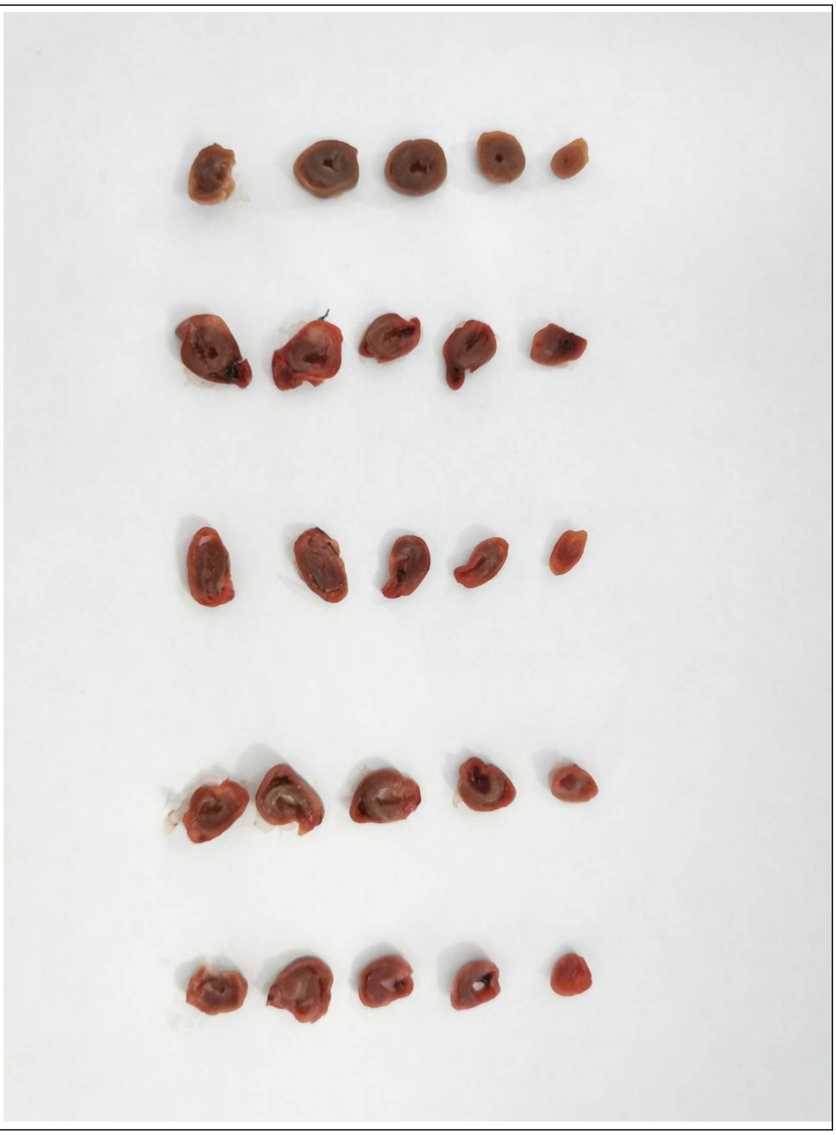
**model**	
**ARS**	
**ARS+miR-29b-3p inhibitor**	
**ARS+miR-29b-3p inhibitor+si-Hmcn 1**	

Western blot

Figure 1D

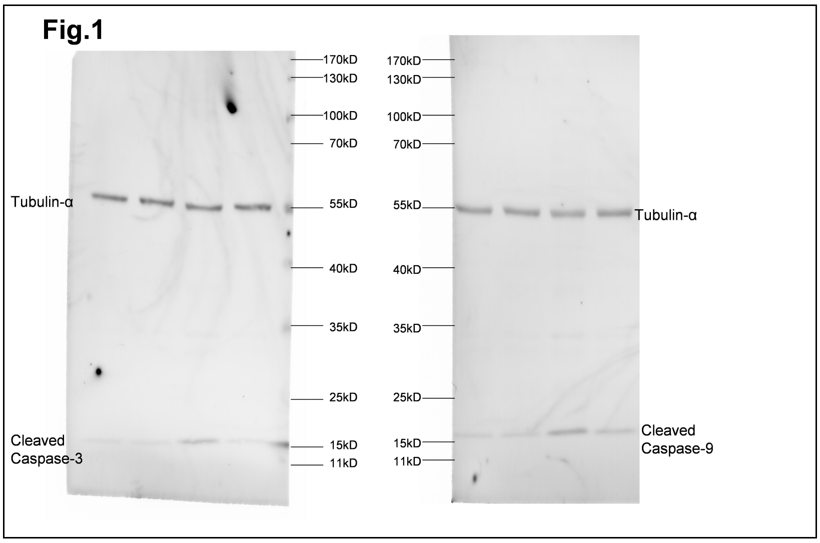

Figure 4C

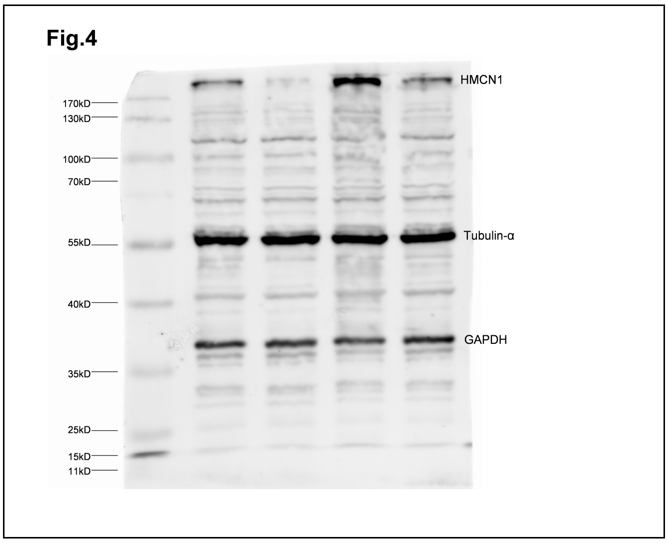

Western blot

Figure 5D

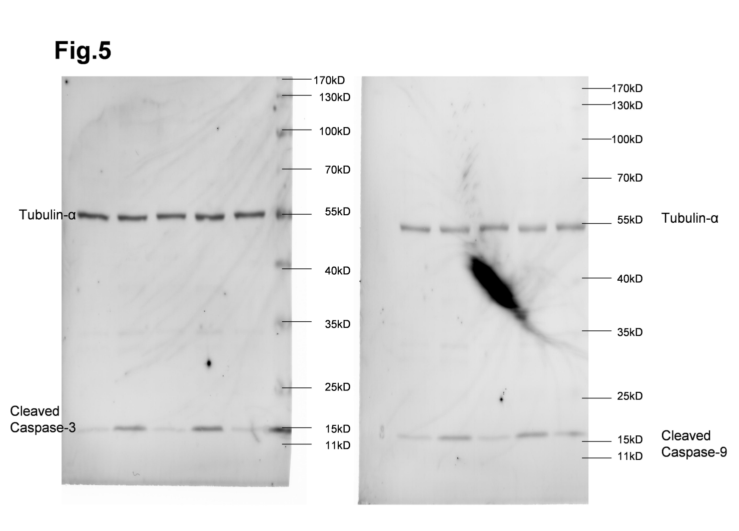

Supplement: Supplementary file 2 [file DataSheet1.PDF]
